# Insurance Denials and Patient Treatment in a Large Academic Radiation Oncology Center

**DOI:** 10.1001/jamanetworkopen.2024.16359

**Published:** 2024-06-12

**Authors:** Jacob Y. Shin, Fumiko Chino, John J. Cuaron, Charles Washington, Margaret Jablonowski, Sean McBride, Daniel R. Gomez

**Affiliations:** 1 Department of Radiation Oncology, Memorial Sloan Kettering Cancer Center, New York, New York; 2 Physician Billing Department, Memorial Sloan Kettering Cancer Center, New York, New York

## Abstract

**Importance:**

Insurance barriers to cancer care can cause significant patient and clinician burden.

**Objective:**

To investigate the association of insurance denial with changes in technique, dose, and time to delivery of radiation oncology treatment.

**Design, Setting, and Participants:**

In this single-institution cohort analysis, data were collected from patients with payer-denied authorization for radiation therapy (RT) from November 1, 2021, to December 8, 2022. Data were analyzed from December 15, 2022, to December 31, 2023.

**Exposure:**

Insurance denial for RT.

**Main Outcomes and Measures:**

Association of these denials with changes in RT technique, dose, and time to treatment delivery was assessed using χ^2^ tests.

**Results:**

A total of 206 cases (118 women [57.3%]; median age, 58 [range, 26-91] years) were identified. Most insurers (199 [96.6%]) were commercial payers, while 7 (3.4%) were Medicare or Medicare Advantage. One hundred sixty-one patients (78.2%) were younger than 65 years. Of 206 cases, 127 (61.7%) were ultimately authorized without any change to the requested RT technique or prescription dose; 56 (27.2%) were authorized after modification to RT technique and/or prescription dose required by the payer. Of 21 cases with required prescription dose change, the median decrease in dose was 24.0 (range, 2.3-51.0) Gy. Of 202 cases (98.1%) with RT delivered, 72 (34.9%) were delayed for a mean (SD) of 7.8 (9.1) days and median of 5 (range, 1-49) days. Four cases (1.9%) ultimately did not receive any authorization, with 3 (1.5%) not undergoing RT, and 1 (0.5%) seeking treatment at another institution.

**Conclusions and Relevance:**

In this cohort study of patients with payer-denied cases, most insurance denials in radiation oncology were ultimately approved on appeal; however, RT technique and/or effectiveness may be compromised by payer-mandated changes. Further investigation and action to recognize the time and financial burdens on clinicians and clinical effects on patients caused by insurance denials of RT is needed.

## Key Points

**Question:**

Is insurance denial associated with radiation oncology treatment?

**Findings:**

In this single-institution cohort analysis of 206 cases, most insurance denials in radiation oncology were ultimately reversed on appeal; however, radiation therapy technique and/or effectiveness may be compromised by payer-mandated changes with resultant delays, reduced dose, or less conformal treatment delivery.

**Meaning:**

These findings suggest that further investigation and action are needed to recognize the time and financial burdens on clinicians as well as the patient outcomes associated with insurance denials of radiation treatments.

## Teaser

This cohort study assesses insurance denials of radiation therapy and their association with changes in radiation technique, dose, and time to treatment delivery among patients with cancer.

## Introduction

The path that physicians face to provide patients with their planned treatment has become increasingly challenging, and prior authorization (PA), a process applied by health insurers requiring clinicians to obtain advanced approval before proposed treatment delivery, has become more common over time.^1^ Health insurers often require PA to verify that a certain cancer treatment is “medically necessary” before it is performed,^2^ and it is not uncommon for health insurance companies to deny claims or not authorize a physician-ordered treatment.^3^ The duration of the appeals process can last days to even months, require additional documents and information, involve scheduling a meeting between the ordering physician and an insurer’s PA peer-to-peer (P2P) specialist, and generate a number of different radiation therapy (RT) plans requiring significant time and effort from the physician and departments of medical radiation physics, dosimetry, treatment devices, and special service.^4,5^ This is especially important for radiation oncology given the significant amount of time and series of complex steps required in the process of care from time of patient consultation to, ultimately, RT delivery and management.

Modern RT incorporates sophisticated planning techniques and on-board imaging to allow the safe and effective delivery of customized precision treatment that benefits patients with improved disease control and fewer adverse effects.^6^ Advanced RT delivery techniques such as intensity-modulated RT (IMRT), stereotactic radiosurgery (SRS), and stereotactic body RT (SBRT) enable a highly conformal technique with sharp dose drop-off to maximize tumor-killing power and minimize off-target radiation exposure to nearby vulnerable tissues. Insurer denials may be a barrier to the growing use^7^ of advanced RT techniques.

This study was designed to assess the downstream sequelae of insurance denials for RT. Specifically, our aim was to evaluate whether these denials were associated with changes in RT technique, radiation dose, and time to treatment delivery.

## Methods

This cohort study was approved by the institutional review board of Memorial Sloan Kettering Cancer Center, New York, New York. Informed consent was waived given that the research involved minimal risk to the participants and data will be deidentified upon completion of the study, with a link to the data kept in a secured database. The study followed the Strengthening the Reporting of Observational Studies in Epidemiology (STROBE) reporting guideline.

### Patients

All patients were seen in a large, quaternary referral, academic cancer center and/or affiliated regional centers. The records of 206 patients (of a total 15 693 cases referred to the billing department) seen between November 1, 2021, and December 8, 2022, were extracted from an institutional registry of patient cases with insurance denials for RT (Table 1). Identification of participant race was performed by the corresponding author (J.Y.S), and the source of the classifications used was the patient electronic health record. Self-identified racial categories included Asian, Black, White, other (including American Indian or Alaska Native, Hispanic, and those classifying themselves as other race), and unknown race. Race was assessed in this study to investigate any possible racial disparities with regard to insurance denial and RT, and data were collected via the patient electronic health record.

**Table 1.** Profile of Cases

Characteristic	Values (N = 206)^a^
Age, y	
Median (range)	58 (26-91)
<65	161 (78.2)
≥65	45 (21.8)
Sex	
Women	118 (57.3)
Men	88 (42.7)
Insurer type	
Commercial	199 (96.6)
Medicare Advantage	4 (1.9)
Medicare	3 (1.5)
Race	
Asian	24 (11.7)
Black	19 (9.2)
White	152 (73.8)
Other^b^	10 (4.9)
Unknown	1 (0.5)
Case type	
Metastatic	141 (68.4)
Definitive	60 (29.1)
Recurrent	5 (2.4)

^a^ Unless otherwise indicated, data are expressed as No. (%) of cases.

^b^ Includes American Indian or Alaska Native, Hispanic, and those classifying themselves as Other.

### Prior Authorization

Prior authorization requests with relevant clinical information were submitted either online (Web Portal) or by telephone to the patient’s insurer prior to treatment initiation. Clinical RT modalities requiring PA included SBRT, IMRT, SRS, 3-dimensional–conformal RT (3D-CRT), 2D-RT (conventional therapy), and brachytherapy. Requested clinical information, including diagnosis codes, disease-specific clinical information, and intended treatment plan (including treatment technique, dose, and number of radiation fractions), were submitted via insurer-provided physician worksheets. Insurer authorization outcomes were faxed (or electronically faxed) to the ordering and/or rendering physician.

### Peer-to-Peer Review

Insurance denials were returned to the cancer center; appeals for further clinical discussion and/or insurer reconsideration, or P2P review, were generally requested within 14 calendar days from the date of determination. For cases still not approved after P2P, a second-level appeal was submitted per discretion of the treating physician using the individual payer-specific process. In combination with or in lieu of P2P, comparison RT plans (for example, IMRT vs 3D-CRT) were submitted for insurer review at the physician’s discretion or at the request of the insurer. Departmental administrative clearance for proceeding with RT without insurer authorization by the time of treatment start date occurred in select cases.

### Variables for Assessment

Measured variables included treatment delay (difference in days between the scheduled and actual RT start dates), RT technique change (ie, SBRT, IMRT, 3D-CRT, and 2D-RT), and prescription dose change (difference in biologically effective dose [BED]). Guideline-concordant treatment was defined as those treatments recommended or suggested by the National Comprehensive Cancer Network, Plymouth Meeting, Pennsylvania.

### Statistical Analysis

Data were analyzed from December 15, 2022, to December 31, 2023. Data on categorical variables were collected and summarized as a series of counts and then arranged in a contingency table. Descriptive statistics to describe quantitative variables used Excel for Microsoft 365 (Microsoft Corporation). We used χ^2^ tests to evaluate whether there was an association between categorical data in rows and columns in a contingency table with SPSS, version 27.0 (IBM Corporation). Two-sided *P* < .05 was considered statistically significant.

## Results

### Patient Characteristics

A total of 206 cases with RT denials were examined. The median age of patients was 58 (range, 26-91) years (Table 1). No patients were younger than 26 years. Fourteen patients (6.8%) were aged 26 to 39 years; 147 (71.4%), aged 40 to 64 years; and 45 (21.8%), 65 years or older. Most patients (199 [96.6%]) had commercial insurance, while 7 (3.4%) had Medicare or Medicare Advantage. Twenty-four patients (11.7%) were Asian; 19 (9.2%), Black; 152 (73.8%), White; 10 (4.9%), Other; and 1 (0.5%), unknown. One hundred eighteen patients (57.3%) were female, and 88 (42.7%) were male. One hundred forty-one cases (68.4%) had metastatic cancer, 60 (29.1%) had a primary tumor, and 5 (2.4%) had recurrent cancer.

### Insurance Appeals

**Figure 1.**
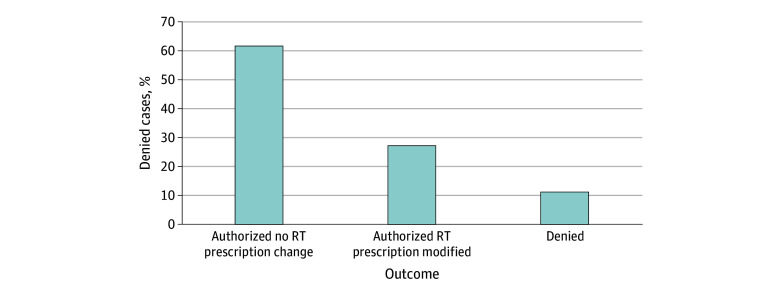
Results of 206 Initially Denied Cases

Of the 206 cases, 127 (61.7%) were ultimately approved by insurance without any change to the requested RT technique or prescription dose after P2P review, second-level appeal, comparison plan submission, employer request, insurance carrier change, and/or external appeal; 56 (27.2%) were authorized after insurer-requested modification to RT technique and/or prescription dose (Figure 1). The remaining 23 cases (11.2%) remained denied by the insurer by the time of RT start.

Of the 56 cases with requested change in RT technique, 20 (35.7% [9.7% of total]) changed from IMRT to 3D-CRT; 16 (28.6% [7.8% of total]), SBRT to 3D-CRT; 15 (26.8% [7.3% of total]), SBRT to IMRT; 4 (7.1% [1.9% of total]), SBRT to 2D-RT; and 1 (1.8% [0.5% of total]), 3D-CRT to 2D-RT. Of the 21 cases (37.5% [10.2% of total]) with a requested change in prescription dose, the median decrease in BED was 24.0 (range, 2.3-51.0) Gy, from a median planned BED of 60.0 (range, 46.9-100.0) Gy to a median delivered BED of 37.5 (range, 14.4-57.6) Gy, using an alpha-beta of 10 (Table 2).

**Table 2.** Prescription Changes in BED Among 21 Cases

Case No.	Initial prescription	Final prescription	BED change^a^
1	37.5 Gy in 15 fractions	36.0 Gy in 15 fractions	−2.3 Gy
2	35.0 Gy in 5 fractions	40.0 Gy in 10 fractions	−3.5 Gy
3	30.0 Gy in 5 fractions	25.0 Gy in 5 fractions	−10.5 Gy
4	35.0 Gy in 5 fractions	30.0 Gy in 5 fractions	−11.5 Gy
5	27.0 Gy in 3 fractions	25.0 Gy in 5 fractions	−13.8 Gy
6	30.0 Gy in 5 fractions	20.0 Gy in 5 fractions	−20.0 Gy
7	35.0 Gy in 5 fractions	25.0 Gy in 5 fractions	−22.0 Gy
8	30.0 Gy in 3 fractions	25.0 Gy in 5 fractions	−22.5 Gy
9	27.0 Gy in 3 fractions	20.0 Gy in 5 fractions	−23.3 Gy
10	24.0 Gy in 1 fraction	36.0 Gy in 6 fractions	−24.0 Gy
11	40.0 Gy in 5 fractions	30.0 Gy in 5 fractions	−24.0 Gy
12	40.0 Gy in 5 fractions	30.0 Gy in 5 fractions	−24.0 Gy
13	40.0 Gy in 5 fractions	30.0 Gy in 5 fractions	−24.0 Gy
14	30.0 Gy in 3 fractions	20.0 Gy in 5 fractions	−32.0 Gy
15	30.0 Gy in 3 fractions	20.0 Gy in 5 fractions	−32.0 Gy
16	30.0 Gy in 3 fractions	20.0 Gy in 5 fractions	−32.0 Gy
17	40.0 Gy in 5 fractions	25.0 Gy in 5 fractions	−34.5 Gy
18	50.0 Gy in 5 fractions	36.0 Gy in 6 fractions	−42.4 Gy
19	30.0 Gy in 3 fractions	8.0 Gy in 1 fraction	−45.6 Gy
20	67.0 Gy in 15 fractions	37.5 Gy in 15 fractions	−51.0 Gy
21	27.0 Gy in 3 fractions	25.0 Gy in 5 fractions	−13.8 Gy

Abbreviation: BED, biologically effective dose.

^a^ Alpha-beta value = 10.

**Figure 2.**
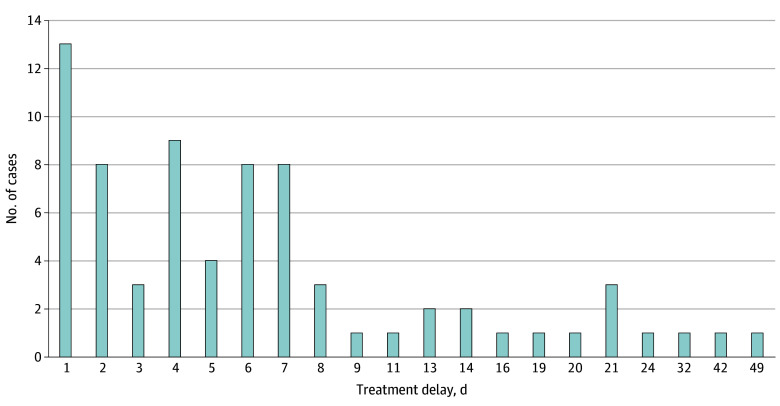
Treatment Delays Among 72 Cases

Of 202 cases (98.1%) in which the patient received treatment, treatment was delayed in 72 (34.9%) for a mean (SD) of 7.8 (9.1) days and a median of 5 (range, 1-49) days (Figure 2). Of the 127 authorized cases with no change in RT plan, treatment was delayed in 43 (20.9%) by a mean (SD) of 7.4 (10.3) days and a median of 4 (range, 1-49) days. No significant differences in treatment delays were observed by age, sex, race, definitive vs metastatic disease, or RT technique.

### Case Mix and Guideline Concordance

Of 60 definitive cases, treatment in 47 (78.3%) was guideline concordant. Ten of the non–guideline-concordant cases were only deemed as such due to requested RT modality and appropriately modified as requested by the insurer (all IMRT to 3D-CRT). The remaining 3 non–guideline-concordant cases were treated in an institutional clinical trial.

Of 61 oligometastatic cases, treatment in 50 (82.0%) was guideline concordant, and non–guideline concordant in 11 (18.0%). The non–guideline-concordant cases were either treated on an institutional protocol for rare histologic findings or for oligometastatic breast cancer after data from NRG Oncology trial BR002 was made known.^8^ Of 64 cases with disseminated disease, 13 (20.3%) had guideline-concordant treatment requests, while 51 (79.7%) did not, with most cases being denied due to SBRT (n = 42) or IMRT (n = 8) requests.

Of the 206 total cases, 9 denied cases (4.4%) included those with oligometastatic brain metastases requesting SRS. Finally, 7 cases (3.4%) involved requesting SBRT or IMRT to previously irradiated sites or those adjacent to prior radiation fields. Of the 5 recurrent cases, all were guideline concordant, with request of IMRT for their respective treatments.

### P2P Review

**Figure 3.**
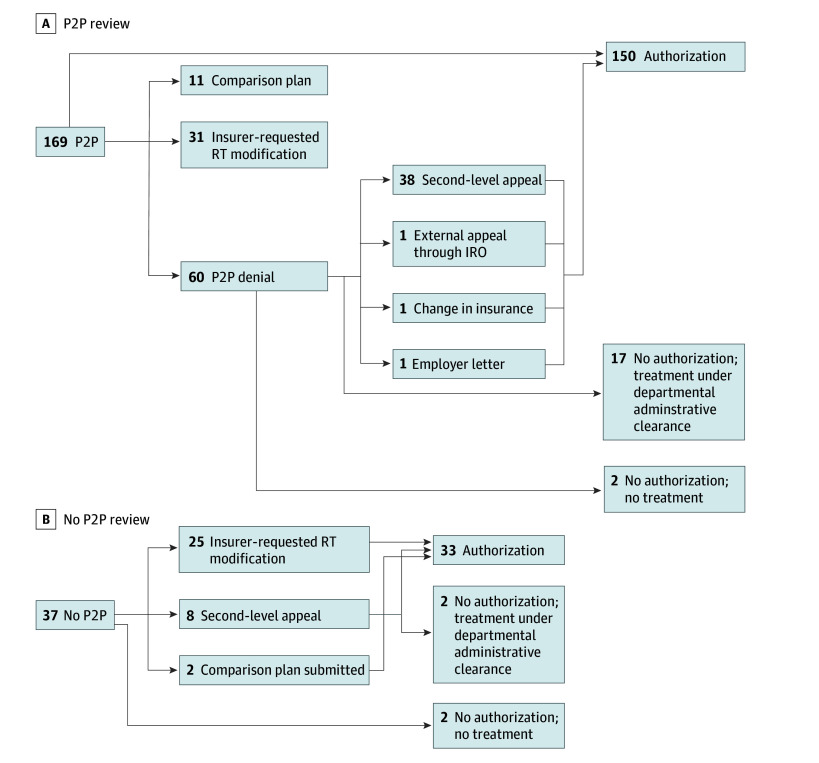
Results of Peer-to-Peer (P2P) and No P2P Review Includes 169 P2P cases and 37 non-P2P cases. IRO indicates independent review organization; RT, radiation therapy.

Of the total 206 cases, P2P review was performed by the treating radiation oncologist in 169 (82.0%) (Figure 3A). Sixty-seven (32.5%) were authorized after P2P alone, 38 (18.4%) after P2P denial but successful second-level appeal, 31 (15.0%) after insurer-requested modifications were completed, 11 (5.3%) after P2P review and a comparison plan (comparison of different RT modalities) submitted, 2 (1.0%) after either an employer letter was written or appeal via an independent review organization was performed, and 1 (0.5%) after patient change in insurance. Seventeen cases (8.3%) ultimately were not authorized by the insurer after P2P review and subsequent processes but proceeded to treatment via departmental administrative clearance. Two cases (1.0%) ultimately did not receive any authorization and did not undergo any RT.

Of the 37 cases (18.0%) in which P2P review was not performed (Figure 3B), 25 (12.1%) were authorized after insurer-request modifications were completed, 6 (2.9%) after successful second-level appeal, and 2 (1.0%) after a comparison plan of different RT modalities was submitted. Two cases (1.0%) ultimately were not authorized by the insurer after second level appeal but proceeded to treatment via departmental administrative clearance. Two cases (1.0%) did not receive any authorization, with one case seeking treatment at another institution and the other case having their RT course cancelled. On multivariate analysis, no significant factors for cases overturned by race, age, sex, stage of disease, or RT technique were observed.

### Clinical Outcomes

Of 60 definitive cases, 6 (10.0%) experienced local failures and 5 (8.3%) died (median follow-up from time of simulation order: 18 [range, 3-26] months). Of 141 metastatic cases, 22 (15.6%) experienced local failures and 78 (55.3%) died (median follow-up: 11 [range, 1-26] months). Of 5 recurrent cases, there were no local failures and no deaths (median follow-up: 26 [range, 3-28] months).

## Discussion

This single-institutional analysis of insurance denials for RT at a large academic cancer center finds that most cases were ultimately approved on appeal. However, RT technique and/or effectiveness may be compromised by payer-mandated changes.

### Prior Authorization

Prior authorization can generate both significant time and financial burdens on physicians and health care practices, leading to potentially enormous organizational costs for institutions nationwide.^9^ For the period 2017 to 2018, Bingham et al^9^ found estimated annual treatment-related PA departmental costs for an academic practice to be $491 989 and on a national level to be $40 125 848. Approved treatments accounted for most of the cost. In our analysis of cases with insurance-denied RT, almost two-thirds of RT plans were approved on appeal without changes; this requires significant effort from clinician teams. Resources and staff time dedicated to insurance appeals could instead be used to focus on patient-specific care, and cancer centers with limited resources and/or staffing shortages may find themselves underresourced to make timely appeals. This may also ultimately affect rural or other disadvantaged populations more discretely.^10^

The PA and appeals process can last days to months prior to treatment authorization, leading to significant delays in radiation oncology care. In a single-institutional analysis of 270 patients treated with curative intent for lung cancer between 2015 and 2018, Salgado et al^11^ found that the median treatment delay was 6.5 days for those requiring P2P vs 1 day for those who did not (*P* = .002), with an increase in frequency of P2P use over the study period. Several studies^11,12^ have demonstrated the negative association between delay of RT and patient clinical outcome. In a systematic review on 46 relevant studies investigating the association between delay in RT and the probability of local control, Huang et al^12^ found that delay in postoperative RT initiation was associated with an increase in 5-year local recurrence rate in breast cancer and head and neck cancer. In our investigation, 72 of 202 cases (34.9%) were delayed for a mean of 7.8 days.

In April 2022, the US Department of Health and Human Services Office of the Inspector General released a report demonstrating that some Medicare Advantage plans were inappropriately delaying and denying medically necessary care in 13% of cases from 2019.^13^ In response, the US House of Representatives passed the Improving Seniors’ Timely Access to Care Act of 2022 in September, which would help establish an electronic PA program, provide a list of services eligible for real-time PA, ensure PA requests are reviewed by qualified medical personnel, and increase transparency around PA requirements and use,^14^ a move applauded by major national oncology associations, including the American Society for Radiation Oncology and American Society of Clinical Oncology.^15,16^ However, the bill was never passed by the US Senate, and as such, never enacted into law by the president.

### Sequelae of Denials

Twenty-one of the total 206 cases (10.2%) in our analysis underwent an insurer-requested change in prescription radiation dose, resulting in a median decrease in BED of 24.0 Gy (median planned BED, 60.0 Gy; median delivered BED, 37.5 Gy). Several studies have shown that lower BED is associated with decreased local tumor control and poorer patient outcomes. In an analysis on 388 patients with 500 metastases after SBRT for liver and lung metastases from colorectal cancer,^17^ higher BED was associated with significantly higher local control rates, and in a retrospective review of 257 patients with stage I non–small cell lung carcinoma (NSCLC), Onishi et al^18^ found that a BED of at least 100 Gy had superior overall survival (*P* < .05) and local control (*P* < .001) rates when compared with conventional RT with lower BED. Clearly, there needs to be scrutiny when insurers are requesting changes to physician-requested prescription dose given the association with patient and disease outcome.

It is important to note that RT-related toxic effects can be a significant factor associated with treatment interruptions and completion, thereby affecting patient clinical outcome and quality of life.^19,20,21^ More than one-quarter of cases in our analysis underwent an insurer-requested change in RT technique that has been associated with change in RT-related toxic effects. A secondary analysis of the NRG Oncology Radiation Therapy Oncology Group 0617 trial found that IMRT was associated with lower rates of severe pneumonitis and cardiac doses when compared with 3D-CRT for chemoradiation treatment with definitive intent in patients with locally advanced NSCLC.^22^ Similar findings of reduced RT-related toxic effects with more conformal RT technique have been demonstrated in head and neck,^23^ brain,^24^ pancreatic,^25,26^ cervical,^27^ and anal^28^ cancers. As such, insurer-related RT treatments associated with lower financial cost may lead to suboptimal patient outcomes and quality of life that may subsequently lead to additional, more costly management in the future.^29^ However, conformal outcome may in certain contexts be similar between SBRT and IMRT.

### Evolving Role of Modern RT in Metastatic Disease

The ongoing developments of immunotherapy and targeted molecular agents have improved the prognosis of those with metastatic disease for different primary cancers.^30,31,32^ As such, RT with a higher BED is being increasingly investigated and used as a means of improving the durability of local control of disease and patient prognosis.^33,34,35,36^ The SABR-COMET (Stereotactic Ablative Radiotherapy for the Comprehensive Treatment of Oligometastatic Tumors) trial^37^ was a phase 2 randomized clinical trial that enrolled 99 patients with a controlled primary malignant neoplasm and with oligometastatic disease (1-5 metastatic lesions), with the most common primary tumor types being breast, lung, colorectal, and prostate. The study demonstrated a significant 5-year overall survival benefit associated with stereotactic ablative radiotherapy with palliative standard-of-care treatment alone. While most of our oligometastatic cases were concordant with the National Comprehensive Cancer Network guidelines regarding oligometastatic disease, a smaller proportion were non–guideline concordant, and additional prospective data are needed to warrant the use of SBRT for different primary cancer sites and their respective subtypes. More recent data from NRG-BR002^8^ suggest no benefit in progression-free survival (PFS) and overall survival when metastasis-directed therapy, including SBRT, is added to standard-of-care treatment in those with oligometastatic breast cancer. A smaller phase 2 randomized clinical trial^38^ demonstrated a PFS benefit associated with SBRT in those with oligoprogressive metastatic NSCLC but did not demonstrate any PFS benefit in those with oligoprogressive metastatic breast cancer.

The role of RT in those with widely metastatic disease continues to evolve as well. Most of our cases with disseminated disease were denied by insurers due to SBRT or IMRT requests. A multicenter randomized phase 2 clinical trial including 78 patients^39^ demonstrated that RT delivered prophylactically to asymptomatic, high-risk bone metastases reduced skeletal-related events and related hospitalizations in patients with widely metastatic solid malignant neoplasms. Notably, the most common RT regimen in the prophylactic RT group was 27 Gy in 3 fractions using SBRT. An open-label, multicenter, randomized, clinical phase 2 to 3 trial of patients with painful spine metastases demonstrated that SBRT at 24 Gy in 2 fractions was superior to conventional external beam RT at a dose of 20 Gy in 5 fractions in improving the complete response rate for pain.^40^ These data suggest that dose-escalated conformal, image-guided RT in carefully selected patients may be appropriate even in the palliative setting and warrant further investigation.

### Limitations

The limitations of this study include the single-institution setting within a restricted geography. Additionally, payer contracts and relations vary by institution, and the ability to overturn case denials may be unrepresentative of other institutions given varying institution types and size, case-mix levels, and patient financial service resources; our department had a 1.3% denial rate during the study period. Notably, at our institution, pediatric radiation oncology cases generally do not get denied, as most insurers waive authorization for RT among patients younger than 18 years. Additionally, the limitations of our current electronic medical record system do not readily allow us to extract demographic and clinicopathologic information from referral requests, including age, race, and stage of disease, on over 15 000 cases to investigate what proportion of cases were denied by these relevant factors and if one group was more likely to be denied than another. Last, longer follow-up is needed to determine whether our findings have a clinical association with outcomes, including palliation, local control, and overall survival.

However, similar challenges with insurance authorization and case denials are faced by numerous hospital departments, and our study is relevant because it analyzes prospectively acquired data representing a snapshot of a greater issue nationwide. To our knowledge, this is the largest study to examine the association of insurance denials within an academic radiation oncology center.

## Conclusions

In this cohort study, most insurance denials in radiation oncology were ultimately approved on appeal; however, RT technique and/or effectiveness may be compromised by payer-mandated changes. These findings suggest a clear need for further investigation and action to recognize the time and financial burdens caused by the increasing use of PA by national insurers and the clinical impact of insurance denials on patient treatment and outcome, to establish more optimal ways to authorize and deliver radiation oncology care in a timely and cost-efficient manner, and to investigate for any possible disparities in insurer treatment authorization outcomes.^41^

## Notes

Supplement.
Data Sharing Statement

## References

[1] Medical Group Management Association (MGMA). Virtually all medical groups say payer prior authorization requirements aren’t improving. March 2, 2022. Accessed June 19, 2023. https://www.mgma.com/mgma-stats/virtually-all-medical-groups-say-payer-prior-authorization-requirements-aren-t-improving

[2] American Cancer Society. Getting medical pre-approval or prior authorization. Updated May 13, 2019. Accessed June 12, 2023. https://www.cancer.org/cancer/financial-insurance-matters/managing-health-insurance/getting-medical-pre-approval-or-prior-authorization.html

[3] American Cancer Society. If your health insurance claim is denied. Updated November 17, 2020. Accessed June 12, 2023. https://www.cancer.org/cancer/financial-insurance-matters/managing-health-insurance/if-your-health-insurance-claim-is-denied.html

[4] Robeznieks A. Facing care denial, oncologist sees 4-week wait for P2P consult. American Medical Association. Updated March 1, 2023. Accessed June 12, 2023. https://www.ama-assn.org/practice-management/prior-authorization/facing-care-denial-oncologist-sees-4-week-wait-p2p-consult

[5] American Society for Radiation Oncology. Process of care. Accessed November 7, 2023. https://www.astro.org/daily-practice/coding/coding-guidance/coding-faqs-and-tips/process-of-care

[6] Bucci MK, Bevan A, Roach M III. Advances in radiation therapy: conventional to 3D, to IMRT, to 4D, and beyond. CA Cancer J Clin. 2005;55(2):117-134. doi:10.3322/canjclin.55.2.117 15761080

[7] Pan HY, Jiang J, Shih YT, Smith BD. Adoption of radiation technology among privately insured nonelderly patients with cancer in the United States, 2008 to 2014: a claims-based analysis. J Am Coll Radiol. 2017;14(8):1027-1033.e2. doi:10.1016/j.jacr.2017.02.040 28408078

[8] Chmura SJ, Winter KA, Woodward WA. NRG-BR002: a phase IIR/III trial of standard of care systemic therapy with or without stereotactic body radiotherapy (SBRT) and/or surgical resection (SR) for newly oligometastatic breast cancer (NCT02364557). J Clin Oncol. 2022;40(16)(suppl):1007. doi:10.1200/JCO.2022.40.16_suppl.1007

[9] Bingham B, Chennupati S, Osmundson EC. Estimating the practice-level and national cost burden of treatment-related prior authorization for academic radiation oncology practices. JCO Oncol Pract. 2022;18(6):e974-e987. doi:10.1200/OP.21.00644 35201904

[10] Kenamond MC, Mourad WF, Randall ME, Kaushal A. No oncology patient left behind: challenges and solutions in rural radiation oncology. Lancet Reg Health Am. 2022;13:100289. doi:10.1016/j.lana.2022.100289 35692288 PMC9170528

[11] Salgado LR, Smith WH, Nehlsen A, . Delays in radiation therapy as a result of insurance peer-to-peer prior authorizations among lung cancer patients. J Radiat Oncol. 2019;8:389-393. doi:10.1007/s13566-019-00409-8

[12] Huang J, Barbera L, Brouwers M, Browman G, Mackillop WJ. Does delay in starting treatment affect the outcomes of radiotherapy? a systematic review. J Clin Oncol. 2003;21(3):555-563. doi:10.1200/JCO.2003.04.171 12560449

[13] Grimm CA. Some Medicare Advantage organization denials of prior authorization requests raise concerns about beneficiary access to medically necessary care. United States Department of Health and Human Services Office of Inspector General. Report in Brief. OEI-09-18-00260. April 27, 2022. Accessed November 15, 2023. https://oig.hhs.gov/oei/reports/OEI-09-18-00260.asp

[14] Improving Seniors’ Timely Access to Care Act of 2022, HR 3173, 117th Cong, 2022. Accessed November 15, 2023. https://www.congress.gov/bill/117th-congress/house-bill/3173/text

[15] American Society of Clinical Oncology. ASCO launches campaign urging Congress to pass prior authorization reform. September 14, 2022. Accessed June 19, 2023. https://society.asco.org/news-initiatives/policy-news-analysis/asco-launches-campaign-urging-congress-pass-prior

[16] American Society for Radiation Oncology. ASTRO applauds House passage of bipartisan bill to reduce prior authorization burden and treatment delays. September 14, 2022. Accessed June 19, 2023. https://www.astro.org/news-and-publications/news-and-media-center/news-releases/2022/astro-applauds-house-passage-of-bipartisan-bill-to-reduce-prior-authorization-burden-and-treatment-d

[17] Klement RJ, Abbasi-Senger N, Adebahr S, . The impact of local control on overall survival after stereotactic body radiotherapy for liver and lung metastases from colorectal cancer: a combined analysis of 388 patients with 500 metastases. BMC Cancer. 2019;19(1):173. doi:10.1186/s12885-019-5362-5 30808323 PMC6390357

[18] Onishi H, Shirato H, Nagata Y, . Hypofractionated stereotactic radiotherapy (HypoFXSRT) for stage I non–small cell lung cancer: updated results of 257 patients in a Japanese multi-institutional study. J Thorac Oncol. 2007;2(7)(suppl 3):S94-S100. doi:10.1097/JTO.0b013e318074de34 17603311

[19] Jeremic B, Shibamoto Y, Milicic B, . Impact of treatment interruptions due to toxicity on outcome of patients with early stage (I/II) non–small-cell lung cancer (NSCLC) treated with hyperfractionated radiation therapy alone. Lung Cancer. 2003;40(3):317-323. doi:10.1016/S0169-5002(03)00078-3 12781431

[20] Lazarev S, Gupta V, Ghiassi-Nejad Z, . Premature discontinuation of curative radiation therapy: insights from head and neck irradiation. Adv Radiat Oncol. 2017;3(1):62-69. doi:10.1016/j.adro.2017.10.006 29556582 PMC5856974

[21] Rosenthal DI, Liu L, Lee JH, . Importance of the treatment package time in surgery and postoperative radiation therapy for squamous carcinoma of the head and neck. Head Neck. 2002;24(2):115-126. doi:10.1002/hed.10038 11891941

[22] Chun SG, Hu C, Choy H, . Impact of intensity-modulated radiation therapy technique for locally advanced non–small-cell lung cancer: a secondary analysis of the NRG Oncology RTOG 0617 randomized clinical trial. J Clin Oncol. 2017;35(1):56-62. doi:10.1200/JCO.2016.69.1378 28034064 PMC5455690

[23] Gupta T, Agarwal J, Jain S, . Three-dimensional conformal radiotherapy (3D-CRT) versus intensity modulated radiation therapy (IMRT) in squamous cell carcinoma of the head and neck: a randomized controlled trial. Radiother Oncol. 2012;104(3):343-348. doi:10.1016/j.radonc.2012.07.001 22853852

[24] MacDonald SM, Ahmad S, Kachris S, . Intensity modulated radiation therapy versus three-dimensional conformal radiation therapy for the treatment of high grade glioma: a dosimetric comparison. J Appl Clin Med Phys. 2007;8(2):47-60. doi:10.1120/jacmp.v8i2.2423 17592465 PMC5722415

[25] Bittner MI, Grosu AL, Brunner TB. Comparison of toxicity after IMRT and 3D-conformal radiotherapy for patients with pancreatic cancer—a systematic review. Radiother Oncol. 2015;114(1):117-121. doi:10.1016/j.radonc.2014.11.043 25497876

[26] Abi Jaoude J, Thunshelle CP, Kouzy R, . Stereotactic versus conventional radiation therapy for patients with pancreatic cancer in the modern era. Adv Radiat Oncol. 2021;6(6):100763. doi:10.1016/j.adro.2021.100763 34934858 PMC8655391

[27] Chopra S, Gupta S, Kannan S, . Late toxicity after adjuvant conventional radiation versus image-guided intensity-modulated radiotherapy for cervical cancer (PARCER): a randomized controlled trial. J Clin Oncol. 2021;39(33):3682-3692. doi:10.1200/JCO.20.02530 34506246

[28] Kachnic LA, Winter KA, Myerson RJ, . Long-term outcomes of NRG Oncology/RTOG 0529: a phase 2 evaluation of dose-painted intensity modulated radiation therapy in combination with 5-fluorouracil and mitomycin-C for the reduction of acute morbidity in anal canal cancer. Int J Radiat Oncol Biol Phys. 2022;112(1):146-157. doi:10.1016/j.ijrobp.2021.08.008 34400269 PMC8688291

[29] Kale HP, Carroll NV. Self-reported financial burden of cancer care and its effect on physical and mental health-related quality of life among US cancer survivors. Cancer. 2016;122(8):283-289. doi:10.1002/cncr.29808 26991528

[30] James ND, de Bono JS, Spears MR, ; STAMPEDE Investigators. Abiraterone for prostate cancer not previously treated with hormone therapy. N Engl J Med. 2017;377(4):338-351. doi:10.1056/NEJMoa1702900 28578639 PMC5533216

[31] Turner NC, Slamon DJ, Ro J, . Overall survival with palbociclib and fulvestrant in advanced breast cancer. N Engl J Med. 2018;379(20):1926-1936. doi:10.1056/NEJMoa1810527 30345905

[32] Gandhi L, Rodríguez-Abreu D, Gadgeel S, ; KEYNOTE-189 Investigators. Pembrolizumab plus chemotherapy in metastatic non–small-cell lung cancer. N Engl J Med. 2018;378(22):2078-2092. doi:10.1056/NEJMoa1801005 29658856

[33] Jung IH, Yoon SM, Kwak J, . High-dose radiotherapy is associated with better local control of bone metastasis from hepatocellular carcinoma. Oncotarget. 2017;8(9):15182-15192. doi:10.18632/oncotarget.14858 28146433 PMC5362477

[34] Machtay M, Bae K, Movsas B, . Higher biologically effective dose of radiotherapy is associated with improved outcomes for locally advanced non–small cell lung carcinoma treated with chemoradiation: an analysis of the Radiation Therapy Oncology Group. Int J Radiat Oncol Biol Phys. 2012;82(1):425-434. doi:10.1016/j.ijrobp.2010.09.004 20980108 PMC5764542

[35] Kumar AMS, Miller J, Hoffer SA, . Postoperative hypofractionated stereotactic brain radiation (HSRT) for resected brain metastases: improved local control with higher BED_10_. J Neurooncol. 2018;139(2):449-454. doi:10.1007/s11060-018-2885-6 29749569

[36] Rades D, Hansen O, Jensen LH, . Radiotherapy for metastatic spinal cord compression with increased radiation doses (RAMSES-01): a prospective multicenter study. BMC Cancer. 2019;19(1):1163. doi:10.1186/s12885-019-6390-x 31783816 PMC6884857

[37] Palma DA, Olson R, Harrow S, . Stereotactic ablative radiotherapy for the comprehensive treatment of oligometastatic cancers: long-term results of the SABR-COMET phase II randomized trial. J Clin Oncol. 2020;38(25):2830-2838. doi:10.1200/JCO.20.00818 32484754 PMC7460150

[38] Tsai CJ, Yang JT, Shaverdian N, ; CURB Study Group. Standard-of-care systemic therapy with or without stereotactic body radiotherapy in patients with oligoprogressive breast cancer or non-small-cell lung cancer (Consolidative Use of Radiotherapy to Block [CURB] oligoprogression): an open-label, randomised, controlled, phase 2 study. Lancet. 2024;403(10422):171-182. doi:10.1016/S0140-6736(23)01857-3 38104577 PMC10880046

[39] Gillespie EF, Yang JC, Mathis NJ, . Prophylactic radiation therapy versus standard of care for patients with high-risk asymptomatic bone metastases: a multicenter, randomized phase II clinical trial. J Clin Oncol. 2024;42(1):38-46. doi:10.1200/JCO.23.00753 37748124 PMC10730067

[40] Sahgal A, Myrehaug SD, Siva S, ; trial investigators. Stereotactic body radiotherapy versus conventional external beam radiotherapy in patients with painful spinal metastases: an open-label, multicentre, randomised, controlled, phase 2/3 trial. Lancet Oncol. 2021;22(7):1023-1033. doi:10.1016/S1470-2045(21)00196-0 34126044

[41] Pendyala P, Goglia AG, Ennis RD. A proposed way forward from the prior authorization crisis in radiation oncology. Appl Radiat Oncol. 2022;(1):7-13. doi:10.37549/ARO1307

