# Supplementary material for: Insurance Denials and Patient Treatment in a Large Academic Radiation Oncology Center
Source: JAMA Netw Open. 2024 Jun 12;7(6):e2416359. doi: 10.1001/jamanetworkopen.2024.16359 (PMC11170304; doi:10.1001/jamanetworkopen.2024.16359)
Supplement: Supplement. — Data Sharing Statement [file jamanetwopen-e2416359-s001.pdf]

Data Sharing Statement

Shin. Insurance Denials and Patient Treatment in a Large Academic Radiation Oncology Center. *JAMA Netw Open*. Published June 12, 2024.
doi:10.1001/jamanetworkopen.2024.16359

Data
Data available: No

Additional Information
Explanation for why data not available: Deidentified data can be made available upon review of request to the corresponding author.
